# Hospitalization and mortality burden of human papillomavirus-associated cancers among men in Ecuador: a nationwide population-based study

**DOI:** 10.3389/fpubh.2026.1980521

**Published:** 2026-10-27

**Authors:** Xavier Sánchez, Ruth Jimbo-Sotomayor, Zira Mendes da Silva, Paula Cristina Pungartnik, Juan Carlos Orengo, Cintia Irene Parellada

**Affiliations:** 1 Centro de Investigación para la Salud en América Latina (CISeAL), Community and Primary Care Research Group Ecuador (CPCRG-E), Pontificia Universidad Católica del Ecuador, Quito, Ecuador; 2 Outcomes Research Latin America, MSD Brazil, São Paulo, Brazil; 3 Outcomes Research Latin America, MSD IA LLC, Guaynabo, Puerto Rico

**Keywords:** Ecuador, hospitalization, HPV-associated cancers, HPV-attributable cancers, human papillomavirus, men, mortality, population-based study

## Abstract

**Introduction:**

Human papillomavirus (HPV)-associated cancers in men contribute substantially to cancer-related morbidity and mortality, yet population-based evidence from Latin America remain limited, particularly in Ecuador.

**Materials and methods:**

Using nationwide hospital discharge and mortality databases from Ecuador for 2015–2019, this retrospective secondary data analysis quantified the burden of HPV-associated cancers among men and estimated the HPV-attributable burden using published site-specific attributable fractions. Crude and age-specific rates were calculated, and age-standardized rates (ASRs) were obtained using the World Health Organization (WHO) World Standard Population.

**Results:**

During 2015–2019, 3,442 hospitalizations due to HPV-associated cancers were recorded (ASR: 9.7 per 100,000 men), with head and neck cancers accounting for 66.4% of hospitalizations, followed by penile cancer (24.0%) and anal cancer (9.6%). Although hospitalization and mortality rates increased markedly with age, more than half of hospitalizations and approximately one-third of deaths from oropharyngeal, penile, and anal cancers occurred among men aged <60 years. Applying published site-specific attributable fractions, 1,002 hospitalizations were estimated to be HPV-attributable (ASR: 2.8 per 100,000 men), with penile cancer contributing the largest share. A total of 575 deaths due to HPV-associated cancers were identified (ASR: 1.7 per 100,000 men), of which 134 were estimated to be HPV-attributable (ASR: 0.4 per 100,000 men). Mortality rates increased progressively with age.

**Conclusion:**

HPV-associated cancers impose a substantial hospitalization and mortality burden among men in Ecuador. Although the highest burden was observed among older adults, a considerable burden was also evident among men aged <60 years. These findings provide a national baseline for surveillance and may inform future evaluations of HPV prevention strategies, alongside efforts to achieve high and equitable HPV vaccination coverage, earlier diagnosis, timely treatment, and improved access to cancer care.

## Introduction

Human papillomavirus (HPV) infection is highly prevalent among men (1). A recent systematic review of global data estimated that approximately one in three men (31%) is infected with at least one genital HPV type, and about one in five (21%) carries a high-risk HPV type (1). Although most infections are transient, persistent HPV infection can lead to anal, penile, and head and neck cancers, including cancers of the oropharynx, larynx, and oral cavity (2, 3). In 2022, HPV was estimated to cause approximately 94,700 cancer cases and 44,000 deaths among men globally (4).

This burden is particularly relevant because, unlike cervical cancer, prevention and early detection options for HPV-related cancers in men remain limited (2, 5, 6). Anal cancer screening is currently limited to selected high-risk populations, and no validated population-based screening strategy exists for head and neck cancers because precursor lesions are poorly defined. Penile cancer is often diagnosed after symptoms at a late stage (7–9). Consequently, HPV vaccination remains central to preventing HPV infection and related disease in men, while limited screening and delayed diagnosis underscore the need for earlier detection and timely cancer care (7–10).

Within Latin America, a growing body of country-level evidence has documented the burden of HPV-associated cancers among men (11–14). Studies from Mexico and Brazil have documented increasing hospitalization burdens for selected HPV-associated cancer sites, particularly anal and oropharyngeal cancers, despite relatively stable overall hospitalization rates (11, 13). In Peru, outpatient consultations exceeded hospitalizations by more than fivefold, suggesting substantial healthcare utilization beyond inpatient care (13). Across studies, the burden was concentrated among middle-aged and older men, reflecting the long latency of HPV-related carcinogenesis (11, 13, 14). In addition, mortality from HPV-attributable head and neck cancers among men was associated with an estimated US$464.2 million in productivity losses across Latin America in 2019 (15). However, despite the growing evidence from neighboring Latin American countries, population-based estimates of hospitalization and mortality due to HPV-associated cancers among men in Ecuador remains limited.

In Ecuador, 9-year-old boys were included in the HPV vaccination program in 2024, a decade after HPV vaccination was introduced for 9-year-old girls through the national immunization program in 2014 (16, 17). Quantifying this burden among men provides a baseline for cancer control planning and future evaluations of male-inclusive HPV vaccination policies in Ecuador. Therefore, this study aimed to characterize the hospitalization and mortality burden of HPV-associated cancers among men in Ecuador during 2015–2019 using nationwide administrative databases. We described patterns by cancer site and age group and estimated the HPV-attributable burden by applying published site-specific attributable fractions (AFs).

## Materials and methods

### Study design, data sources, and population

We conducted an observational, nationwide, retrospective secondary data analysis of hospitalizations and deaths due to HPV-associated cancers among men in Ecuador from January 1, 2015, to December 31, 2019. The study period was restricted *a priori* to 2015–2019 to establish a pre-pandemic baseline for HPV-associated cancer hospitalizations and mortality. Data from 2020 onward were not included because pandemic-related changes in healthcare utilization, diagnostic activity, hospital admissions, and mortality registration could affect temporal comparability.

Data were obtained from two publicly available nationwide databases maintained by Ecuador’s National Institute of Statistics and Censuses (*Instituto Nacional de Estadística y Censos* [INEC]) (18, 19). These databases contain only de-identified records. Hospitalization data were extracted from the Statistical Registry of Hospital Beds and Discharges (*Registro Estadístico de Camas y Egresos Hospitalarios*), which records hospital discharge events reported by public and private facilities nationwide (18). The unit of observation in this database is the discharge event rather than the individual patient. Because patient-level identifiers are not available, linkage of multiple admissions for the same individual was not possible; therefore, hospitalization estimates should be interpreted as healthcare utilization events rather than numbers of individual patients or incident cancer cases.

Mortality data were obtained from the Statistical Registry of General Deaths (Registro Estadístico de Defunciones Generales) (19), a nationwide mortality database maintained by INEC as part of Ecuador’s civil registration and vital statistics system. Death registration has operated for decades, transitioning progressively from paper-based to electronic systems as part of national modernization efforts (20, 21). Previous assessments have identified geographical variation in the completeness and quality of mortality registration in Ecuador (21).

### Outcomes and definitions

The primary outcomes were the counts and rates of hospitalizations and deaths due to HPV-associated cancers among men, stratified by cancer site (anal, penile, oral cavity, oropharyngeal, and laryngeal cancers) and age group (overall, <30, 30–59 and ≥60 years).

HPV-associated cancers were defined as invasive cancers at anatomical sites where HPV is known to contribute to carcinogenesis and where HPV DNA is frequently detected, regardless of individual tumor HPV status, consistent with previously published population-based studies (11, 13, 14, 22). Accordingly, HPV-associated cancers include all cancers occurring at these anatomical sites. HPV-attributable cancers represent the estimated subset of HPV-associated cancers that may be causally attributed to HPV infection.

HPV-associated events were identified using International Classification of Diseases, 10th Revision (ICD-10) codes assigned as the principal discharge diagnosis for hospitalizations or as the underlying cause of death in mortality records: anal cancer (C21.0-C21.8), penile cancer (C60.0-C60.9), oral cavity cancer (C02.0-C02.3, C03.0-C03.9, C04.0-C04.9, C05.0, C06.0-C06.9), oropharyngeal cancer (C01, C02.4, C05.1, C05.2, C09.0-C09.9, C10.0, C10.2-C10.9), and laryngeal cancer (C32.0-C32.9) (Supplementary Table S1). Head and neck cancers were additionally examined as a combined category comprising oral cavity, oropharyngeal, and laryngeal cancers.

To estimate the HPV-attributable burden, published site-specific AFs were applied to the observed numbers of HPV-associated cancer hospitalizations and deaths. Therefore, the resulting estimates were derived from published AFs rather than direct measurements of tumor HPV status. Site-specific AFs were selected from the literature, prioritizing estimates derived from Latin American populations using standardized HPV detection methods when available. For head and neck cancers, AFs derived from South American tumor HPV DNA and p16 data were 6.3% for oral cavity cancer, 21.2% for oropharyngeal cancer, and 4.5% for laryngeal cancer (23). Published global AFs were applied to anal cancer (100%) and penile cancer (51%) (24, 25). Site-specific AFs were applied uniformly across age groups and calendar years because age- and year-specific estimates were unavailable. Additional details on the selected AFs and supporting references are provided in Supplementary Table S2.

### Statistical analysis

Events were aggregated over the five-year study period and summarized by cancer site and age group (overall, <30, 30–59, and ≥60 years). Descriptive analyses included event counts, proportions, and crude, age-specific, and age standardized rates for hospitalization and mortality.

Crude and age-specific average rates were calculated by dividing the total number of events recorded during 2015–2019 by the sum of the corresponding annual male population estimates over the same period and were expressed per 100,000 men (26). Age-standardized rates (ASRs) were calculated using the direct standardization method with the World Health Organization (WHO) World Standard Population (2000–2025) as the reference population to facilitate comparisons across populations with different age structures (27). HPV-attributable counts derived using the published site-specific AFs were used to calculate the corresponding crude, age-specific, and age-standardized rates.

Data cleaning and preparation were performed using Python version 3.7.7 (Python Software Foundation); proportions and rates were calculated using Microsoft Excel (Microsoft Corp.).

Although the study used publicly available databases containing only de-identified records, the protocol was submitted for ethics review as an additional measure of ethical oversight and research governance. The protocol was approved by the Research Ethics Committee on Human Subjects of Universidad San Francisco de Quito (CEISH-USFQ), Ecuador, on May 24, 2022 (Approval No. P22-018E; Protocol Reference No. IE02-EX045-2022-CEISH-USFQ). The research was conducted in accordance with the ethical principles of the Declaration of Helsinki, and reporting followed the Strengthening the Reporting of Observational Studies in Epidemiology (STROBE) guidelines (28).

Artificial intelligence (AI)-based language-editing tools were used to support language refinement and structural editing during manuscript preparation. All scientific content, interpretation, and final editorial decisions were reviewed and approved by the authors, who take full responsibility for the manuscript.

## Results

### HPV-associated cancer hospitalizations

Over the five-year period from 2015 to 2019, 3,442 hospitalizations for HPV-associated cancers were recorded among men, averaging approximately 688 per year. The overall crude rate and ASR were 8.2 and 9.7 hospitalizations per 100,000 men, respectively (Table 1). Two-thirds of hospitalizations were due to head and neck cancers (66.4%), with penile cancer (24.0%) and anal cancer (9.6%) comprising the remainder. Within the head and neck category, laryngeal cancer accounted for the largest proportion of hospitalizations (44.3%), followed by oropharyngeal cancer (36.6%) and oral cavity cancer (19.2%). A steep age gradient was observed, with men aged ≥60 years accounting for 54.3% of all HPV-associated cancer hospitalizations and having an age-specific rate of 47.6 per 100,000 men. The proportion of hospitalizations occurring among men aged ≥60 years was highest for laryngeal cancer (69.4%), followed by oral cavity cancer (63.0%). Among men aged 30–59 years, the age-specific hospitalization rate was 9.5 per 100,000 men, and this age group accounted for 50.1% of oropharyngeal, 45.1% of penile, and 43.5% of anal cancer hospitalizations. Hospitalizations among men aged <30 years accounted for 6.9% of the overall burden and were proportionally highest for anal cancer (14.3%) and oral cavity cancer (13.2%) (Figures 1, 2).

**Table 1:** Counts and rates of hospitalizations for HPV-associated and HPV-attributable cancers among men in Ecuador, 2015–2019.

Cancer site	All ages			<30		30–59		≥60	
	*n*	Crude Rate	ASR	*n*	Rate	*n*	Rate	*n*	Rate
HPV-associated cancers									
Overall	3,442	8.2	9.7	237	1.0	1,337	9.5	1868	47.6
Anal cancer	329	0.8	0.9	47	0.2	143	1.0	139	3.5
Penile cancer	827	2.0	2.3	72	0.3	373	2.6	382	9.7
Head and neck cancers	2,286	5.5	6.5	118	0.5	821	5.8	1,347	34.3
Oral cavity cancer	438	1.0	1.2	58	0.2	104	0.7	276	7.0
Oropharyngeal cancer	836	2.0	2.3	48	0.2	419	3.0	369	9.4
Laryngeal cancer	1,012	2.4	3.0	12	0.1	298	2.1	702	17.9
HPV-attributable cancers									
Overall	1,002	2.4	2.8	99	0.4	442	3.1	461	11.8
Anal cancer	329	0.8	0.9	47	0.2	143	1.0	139	3.5
Penile cancer	422	1.0	1.2	37	0.2	190	1.3	195	5.0
Head and neck cancers	251	0.6	0.7	15	0.1	109	0.8	127	3.2
Oral cavity cancer	28	0.1	0.1	4	<0.1	7	<0.1	17	0.4
Oropharyngeal cancer	177	0.4	0.5	10	<0.1	89	0.6	78	2.0
Laryngeal cancer	46	0.1	0.1	1	<0.1	13	0.1	32	0.8

HPV, human papillomavirus; n, total number of hospitalizations, 2015–2019; ASR, age-standardized rate. Head and neck cancers are presented as an aggregated category comprising oral cavity, oropharyngeal, and laryngeal cancers. Rates are expressed per 100,000 men and age-standardized to the WHO World Standard Population (2000–2025). HPV-attributable counts were derived by applying site-specific attributable fractions.

**Figure 1:**
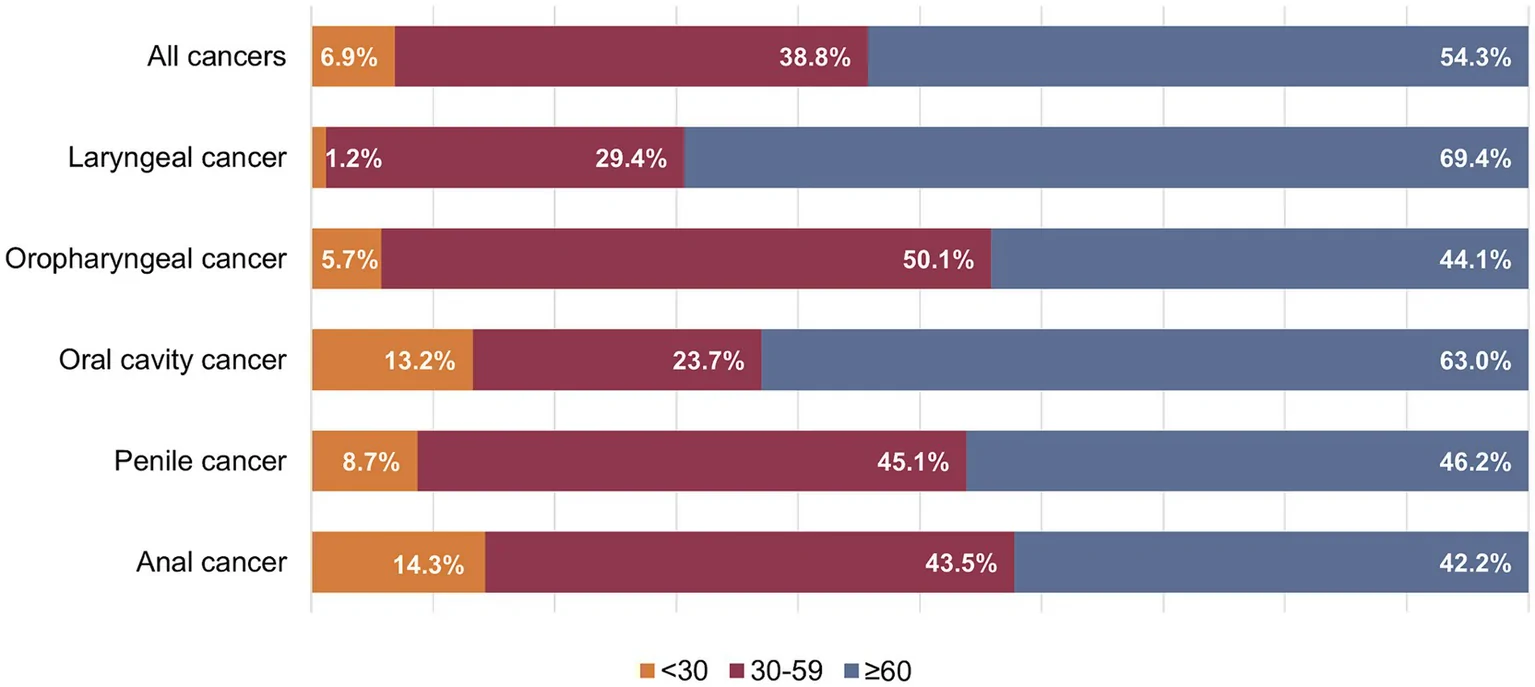
Proportion of hospitalizations for HPV-associated cancers among men in Ecuador, 2015–2019. Colored bar segments represent age-groups <30, 30–59, and ≥60 years and show the percentage distribution of hospitalizations within each cancer site among men in Ecuador, 2015–2019.

**Figure 2:**
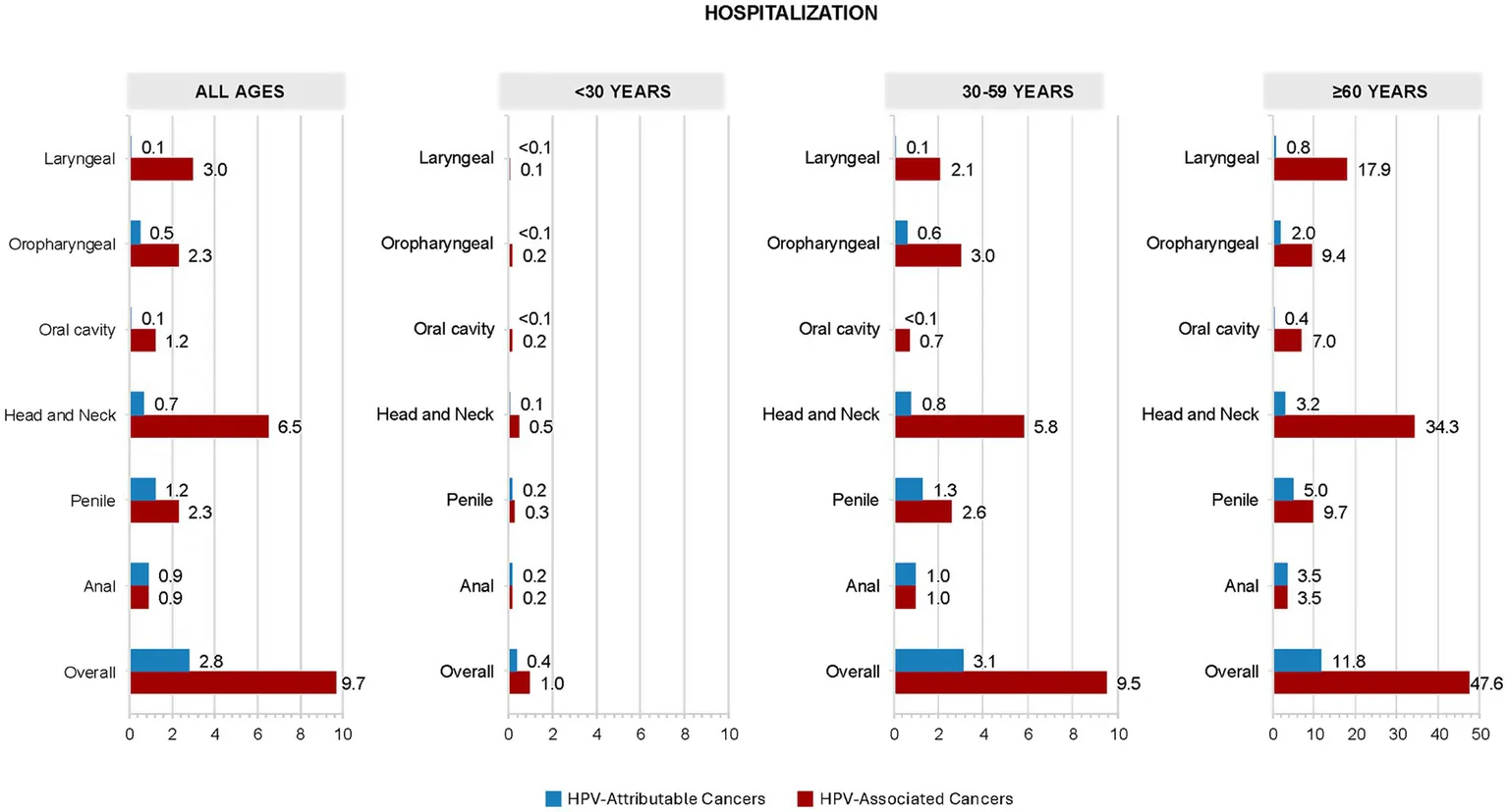
Hospitalization rates for HPV-associated and HPV-attributable cancers among men in Ecuador, 2015–2019. Rates are expressed per 100,000 men in Ecuador, 2015–2019. Head and neck cancers comprise oral cavity, oropharyngeal, and laryngeal cancers. HPV-attributable estimates were obtained by applying published site-specific attributable fractions to HPV-associated hospitalization counts. Rates <0.05 per 100,000 are displayed as <0.1.

### HPV-attributable cancer hospitalizations

After applying published site-specific AFs, 1,002 hospitalizations were estimated to be HPV-attributable, corresponding to 29.1% of all HPV-associated cancer hospitalizations. The estimated crude rate and ASR were 2.4 and 2.8 hospitalizations per 100,000 men, respectively (Table 1; Figure 2). Penile cancer accounted for the largest share of HPV-attributable hospitalizations (42.1%), followed by anal cancer (32.8%) and head and neck cancers (25.0%). Within the head and neck cancer category, oropharyngeal cancer accounted for 70.5% of estimated HPV-attributable hospitalizations. The age distribution of HPV-attributable hospitalizations broadly mirrored that observed for HPV-associated cancer hospitalizations.

### HPV-associated cancer mortality

During the study period, 575 deaths due to HPV-associated cancers were recorded, averaging 115 deaths per year. The crude and age-standardized mortality rates were 1.4 and 1.7 per 100,000 men, respectively (Table 2). Head and neck cancers accounted for the largest share of deaths (71.1%), followed by penile cancer (23.3%) and anal cancer (5.6%). Within the head and neck cancer category, laryngeal cancer was the primary contributor (63.6%), followed by oropharyngeal cancer (21.0%) and oral cavity cancer (15.4%).

**Table 2:** Counts and rates of deaths for HPV-associated and HPV-attributable cancers among men in Ecuador, 2015–2019.

Cancer site	All ages			<30		30–59		≥60	
	*n*	Crude rate	ASR	*n*	Rate	*n*	Rate	*n*	Rate
HPV-associated cancers									
Overall	575	1.4	1.7	12	0.1	145	1.0	418	10.7
Anal cancer	32	0.1	0.1	1	<0.1	9	0.1	22	0.6
Penile cancer	134	0.3	0.4	7	<0.1	46	0.3	81	2.1
Head and neck cancers	409	1.0	1.2	4	<0.1	90	0.6	315	8.0
Oral cavity cancer	63	0.1	0.2	2	<0.1	12	0.1	49	1.2
Oropharyngeal cancer	86	0.2	0.2	1	<0.1	32	0.2	53	1.4
Laryngeal cancer	260	0.6	0.8	1	<0.1	46	0.3	213	5.4
HPV-attributable cancers									
Overall	134	0.3	0.4	5	<0.1	42	0.3	87	2.2
Anal cancer	32	0.1	0.1	1	<0.1	9	0.1	22	0.6
Penile cancer	68	0.2	0.2	4	<0.1	23	0.2	41	1.0
Head and neck cancers	34	0.1	0.1	0	0.0	10	<0.1	24	0.6
Oral cavity cancer	4	0.0	<0.1	0	0.0	1	<0.1	3	0.1
Oropharyngeal cancer	18	<0.1	0.1	0	0.0	7	<0.1	11	0.3
Laryngeal cancer	12	<0.1	<0.1	0	0.0	2	<0.1	10	0.3

HPV, human papillomavirus; n, total number of deaths, 2015–2019; ASR, age-standardized rate. Head and neck cancers comprise oral cavity, oropharyngeal, and laryngeal cancers. Rates <0.05 per 100,000 are displayed as <0.1. Rates are expressed per 100,000 men and age-standardized to the WHO World Standard Population (2000–2025). HPV-attributable counts were derived by applying site-specific attributable fractions.

Mortality rates increased progressively with age, with men aged ≥60 years accounting for 72.7% of all HPV-associated cancer deaths. The highest age-specific mortality rate was observed for head and neck cancers in this age group (8.0 per 100,000 men). Although mortality rates increased sharply with age, a substantial proportion of deaths occurred before age 60, with men aged 30–59 years accounting for 37.2% of oropharyngeal cancer deaths and 34.3% of penile cancer deaths. Deaths were also recorded among men aged <30 years, representing 2.1% of all HPV-associated cancer deaths. Overall, 39.6% of penile, 38.4% of oropharyngeal, and 31.3% of anal cancer deaths occurred before age 60 (Figures 3, 4).

**Figure 3:**
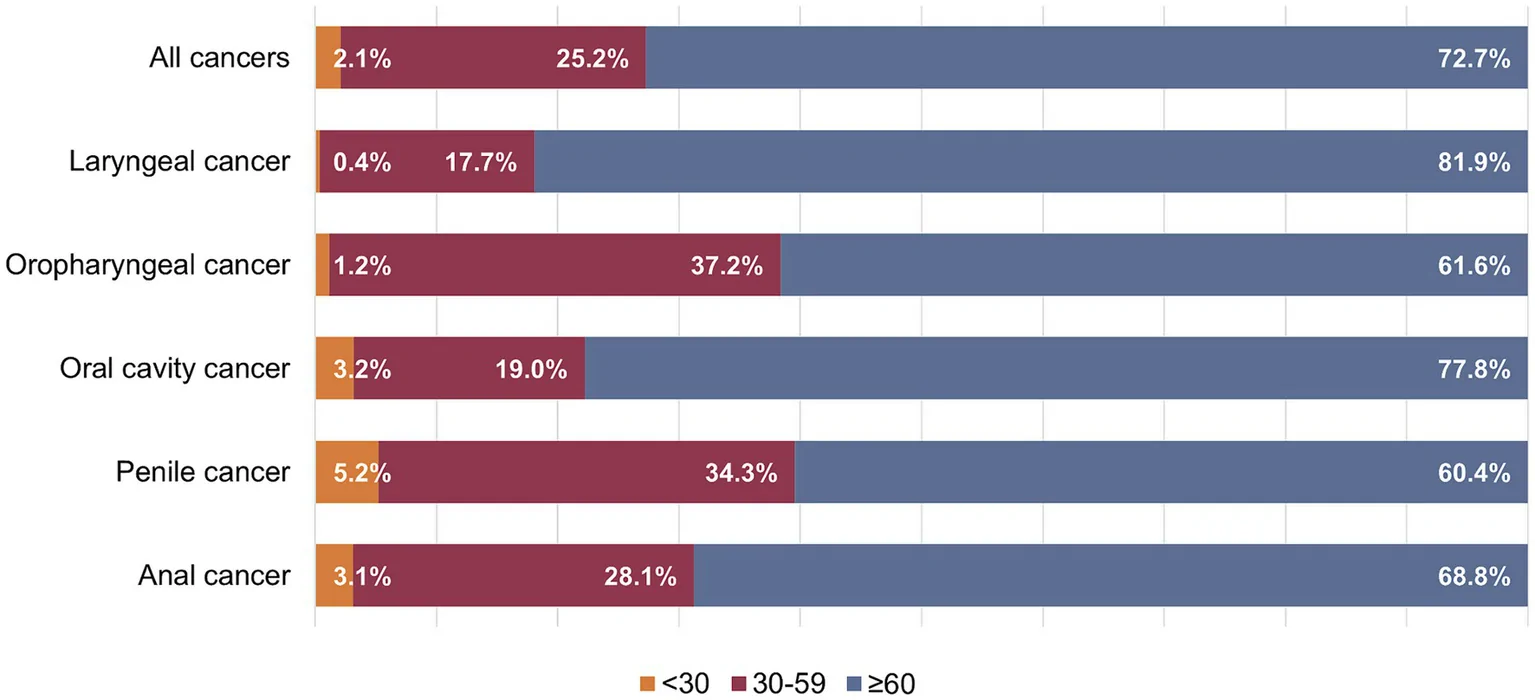
Proportion of deaths for HPV-associated cancers among men in Ecuador, 2015–2019. Colored bar segments represent age-groups <30, 30–59, and ≥60 years and show the percentage distribution of deaths within each cancer site among men in Ecuador, 2015–2019.

**Figure 4:**
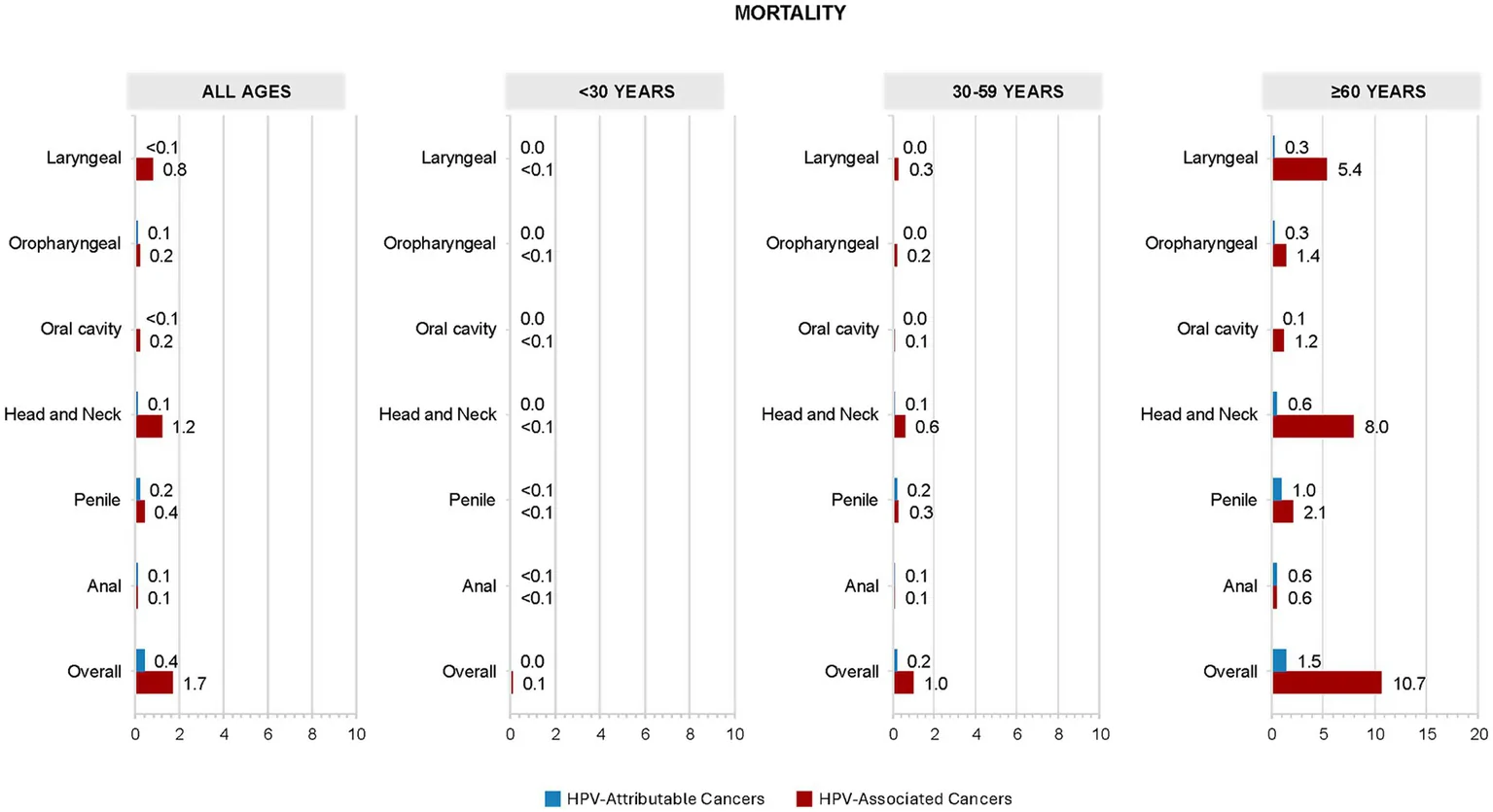
Mortality rates for HPV-associated and HPV-attributable cancers among men in Ecuador, 2015–2019. Rates are expressed per 100,000 men in Ecuador, 2015–2019. Head and neck cancers comprise oral cavity, oropharyngeal, and laryngeal cancers. HPV-attributable estimates were obtained by applying published site-specific attributable fractions to HPV-associated death counts. Rates <0.05 per 100,000 are displayed as <0.1.

### HPV-attributable cancer mortality

After applying published site-specific AFs, 134 deaths were estimated to be HPV-attributable, corresponding to 23.3% of all HPV-associated cancer deaths. The estimated crude and age-standardized mortality rates were 0.3 and 0.4 per 100,000 men, respectively (Table 2; Figure 4). Penile cancer accounted for the largest share of HPV-attributable deaths (50.7%), followed by head and neck cancers (25.4%) and anal cancer (23.9%). The age distribution of HPV-attributable mortality was similar to that observed for HPV-associated mortality.

## Discussion

This nationwide study characterized the hospitalization and mortality burden of HPV-associated cancers among men in Ecuador and estimated the proportion attributable to HPV. Over a five-year period, 3,442 hospitalizations and 575 deaths due to HPV-associated cancers were recorded, of which approximately one in three hospitalizations and one in four deaths were estimated to be HPV-attributable. Head and neck cancers accounted for the largest share of HPV-associated hospitalizations and deaths, whereas penile cancer was the leading contributor to the estimated HPV-attributable burden. By extending the growing body of evidence from Latin America (11, 13–15, 29–34), this study contributes country-specific estimates that support surveillance, cancer-control planning, and future evaluations of Ecuador’s gender-inclusive HPV vaccination policies.

Consistent with findings from Peru, Mexico, and Brazil (11, 13, 14), men aged ≥60 years accounted for 54.3% of hospitalizations and 72.7% of deaths in Ecuador. Nonetheless, the age distribution varied by cancer site: more than half of hospitalizations for oropharyngeal, penile, and anal cancers occurred before age 60, as did approximately one-third of deaths from these cancers. This heterogeneity highlights the importance of examining age alongside demographic, clinical, and healthcare-related factors to better characterize the populations affected. This burden observed before age 60 has implications beyond clinical outcomes. A recent study from Brazil and Mexico estimated approximately two decades of life lost per HPV-attributable cancer death among men (35). Complementing these findings, a modeling study across Latin America, which included Ecuador, estimated an average of 22 years of life lost per HPV-attributable head and neck cancer death and productivity losses of US$2.9 million for Ecuador in 2019 (15).

After applying site-specific HPV-attributable fractions (AFs), penile and anal cancers each accounted for a larger share of the HPV-attributable burden than head and neck cancers. This pattern reflects the multifactorial etiology of head and neck cancers, with the relative contribution of HPV, tobacco, and alcohol varying across anatomical sites and populations (2, 23). Nevertheless, HPV has become an increasingly important contributor to oropharyngeal cancer in several high-income countries, while studies from Mexico and Brazil have documented an increasing burden of oropharyngeal cancer in men and younger populations, respectively (11, 30, 36, 37). A recent multinational study including men from Brazil and Mexico demonstrated that oral HPV acquisition occurs throughout adulthood (38). In our analysis, oropharyngeal cancer accounted for the largest share of HPV-attributable head and neck cancer hospitalizations in Ecuador, with 55.9% of oropharyngeal cancer hospitalizations and 38.4% of oropharyngeal cancer deaths occurring among men aged <60 years. Together, these findings support the need for sustained prevention efforts across the life course.

Penile cancer accounted for approximately four in ten estimated HPV-attributable hospitalizations and one in two estimated HPV-attributable deaths in Ecuador. For HPV-associated penile cancer, the age-standardized hospitalization rate was 2.3 per 100,000 men, compared with 0.7 in Peru, 1.7 in Mexico, and 2.0 in Brazil (11, 13, 14). The comparatively high burden observed in Ecuador is consistent with evidence that the disease disproportionately affects populations experiencing socioeconomic disadvantage, limited access to sanitation, and lower circumcision rates (32, 33, 39). Delayed healthcare seeking may further contribute to this burden, with an average delay of approximately 19 months reported in the region (33).

Although anal cancer represented a smaller proportion of hospitalizations (9.6%) and deaths (5.6%), it showed a relatively younger age distribution, with 57.8% of hospitalizations occurring among men aged <60 years. Ecuador’s age-standardized hospitalization rate for HPV-associated anal cancer was 0.9 per 100,000 men, compared with 0.1 in Peru, 0.7 in Mexico, and 1.5 in Brazil (11, 13, 14). However, population-level estimates may not fully reflect the burden among high-risk groups, as anal cancer incidence has been reported to reach 59.1 per 100,000 person-years among men who have sex with men living with HIV in Latin America (31). The substantial burden observed before age 60 in Ecuador, together with the elevated anal cancer incidence reported among men who have sex with men living with HIV in Latin America, reinforces the importance of HPV vaccination and guideline-based anal cancer screening for eligible high-risk groups (7, 31, 40).

The higher age-standardized rates relative to crude rates reflect Ecuador’s relatively young population. Together with the concentration of hospitalizations and deaths among older adults, this finding highlights the importance of continued surveillance as the population ages. Within the available Latin American evidence, Ecuador exhibited an age-standardized HPV-associated hospitalization rate of 9.7 per 100,000 men, which was higher than the rates reported in Peru (1.6) and Mexico (7.8), but lower than that reported in Brazil (24.5) (11, 13, 14). Cross-country differences in hospitalizations may reflect variation in demographic structure, healthcare access, coding practices, completeness of registry data, case ascertainment, and underlying epidemiology.

Because the study period preceded routine HPV vaccination for boys in Ecuador, these findings provide a baseline for assessing future changes in hospitalization and mortality burden. The inclusion of 9-year-old boys in Ecuador’s HPV vaccination program in 2024 aligns with PAHO’s Expanded Program on Immunization (EPI) recommendations to close coverage gaps and consider vaccination of both girls and boys (16, 41, 42). Its population impact will depend on achieving and sustaining high and equitable HPV vaccination coverage, including through catch-up strategies for eligible unvaccinated individuals, and addressing barriers to awareness, access, and implementation (34). A study conducted in Ecuador identified limited awareness of the relationship between HPV and cancer in men, together with informational and health-system barriers to vaccination, while regional evidence indicates that vaccine acceptance is influenced by knowledge, safety concerns, socioeconomic factors, healthcare recommendations, and access (43, 44). Gender-inclusive vaccination policies should therefore be supported by effective communication, community engagement, and context-specific delivery strategies. Because the current cancer burden largely reflects infections acquired before vaccine availability, prevention must be complemented by early diagnosis, timely treatment, and access to specialized oncologic services.

This study has limitations inherent to analyses of administrative databases. First, hospitalization records represent discharge events rather than unique individuals and may include repeated admissions. Second, mortality estimates may be affected by the completeness and accuracy of cause-of-death reporting. Third, the absence of outpatient data may have led to underestimation of the overall burden, particularly because previous Latin American studies have suggested that HPV-associated cancers may generate substantial healthcare utilization outside the inpatient setting. Finally, AFs were assumed to remain constant across age groups and calendar years because more granular estimates were unavailable. Accordingly, the HPV-attributable estimates should be interpreted as modeled estimates derived from published site-specific AFs rather than direct measurements of tumor HPV status. The available administrative data also lacked the individual-level covariates needed for multivariable analyses.

A major strength of this study is the use of nationwide administrative databases encompassing both public and private settings over a five-year period, enabling a comprehensive characterization of hospitalization and mortality patterns of HPV-associated cancers among men in Ecuador. Although hospitalization data do not represent cancer incidence, they capture severe disease requiring inpatient care and therefore provide important information regarding healthcare system burden. To our knowledge, this is the first nationwide study to simultaneously characterize hospitalization burden, mortality burden, and estimated HPV-attributable burden among men in Ecuador. The use of site-specific AFs facilitates comparisons with similar burden-of-disease studies conducted in Latin America.

## Conclusion

HPV-associated cancers continue to impose a substantial hospitalization and mortality burden among men in Ecuador, with a meaningful proportion of events estimated to be attributable to HPV. Although the burden was concentrated among older adults, substantial proportions of hospitalizations and deaths at selected cancer sites occurred before age 60, indicating important health and societal consequences across adulthood. Given the long natural history of HPV-related carcinogenesis and the absence of validated population-based screening strategies for most HPV-related cancers in men, reducing the future burden will require sustained efforts to achieve and maintain high and equitable vaccination coverage. These preventive efforts should be complemented by earlier diagnosis, timely treatment, and equitable access to cancer care for individuals already affected by or at risk of HPV-related cancers. These findings provide an important national baseline for future evaluation of Ecuador’s gender-inclusive HPV vaccination strategy and may inform vaccination policies, resource allocation, surveillance, and broader cancer-control strategies in Ecuador.

## Data Availability

The datasets analyzed in this study are publicly available from the Instituto Nacional de Estadística y Censos (INEC), Ecuador, and can be accessed through the official INEC website: https://www.ecuadorencifras.gob.ec/institucional/home/.
