# Supplementary material for: Hospitalization and mortality burden of human papillomavirus-associated cancers among men in Ecuador: a nationwide population-based study
Source: Front Public Health. 2026 Oct 27;14:1980521. doi: 10.3389/fpubh.2026.1980521 (PMC13748293; doi:10.3389/fpubh.2026.1980521)
Supplement: Supplementary file 1 [file Table_1.docx]

Supplemental Table S1. Cancer by anatomic site and corresponding International Classification of Diseases (tenth revision) codes used for case identification in Ecuadorian national health databases

| Condition | | ICD-10 codes | |
| --- | --- | --- | --- |
| **ANOGENITAL CANCERS** | | |  |
| Anal Cancer | C21 Malignant neoplasm of anus and anal canal | |  |
|  | C21.0 Malignant neoplasm of anus, unspecified | |  |
|  | C21.1 Malignant neoplasm of anal canal | |  |
|  | C21.2 Malignant neoplasm of cloacogenic zone | |  |
|  | C21.8 Malignant neoplasm of overlapping sites of rectum, anus and anal canal | |  |
| Penile Cancer | C60 Malignant neoplasm of penis | |  |
|  | C60.0 Malignant neoplasm: Prepuce. | |  |
|  | C60.1 Malignant neoplasm: Glans penis | |  |
|  | C60.2 Malignant neoplasm: Body of penis | |  |
|  | C60.8 Malignant neoplasm: Overlapping lesion of penis | |  |
|  | C60.9 Malignant neoplasm: Penis, unspecified | |  |
| **HEAD AND NECK CANCERS** | | |  |
| Laryngeal Cancer | C32 Malignant neoplasm of larynx | |  |
|  | C32.0 Malignant neoplasm of glottis | |  |
|  | C32.1 Malignant neoplasm of supraglottis | |  |
|  | C32.2 Malignant neoplasm of subglottis | |  |
|  | C32.3 Malignant neoplasm of laryngeal cartilage | |  |
|  | C32.8 Malignant neoplasm of overlapping sites of larynx | |  |
|  | C32.9 Malignant neoplasm of larynx, unspecified | |  |
| Oral cavity Cancer | C02.0 Malignant neoplasm of dorsal surface of tongue | |  |
|  | C02.1 Malignant neoplasm of border of tongue | |  |
|  | C02.2 Malignant neoplasm of ventral surface of tongue | |  |
|  | C02.3 Malignant neoplasm of anterior two-thirds of tongue, part unspecified | |  |
|  | C03 Malignant neoplasm of gum | |  |
|  | C03.0 Malignant neoplasm of upper gum | |  |
|  | C03.1 Malignant neoplasm of lower gum | |  |
|  | C03.9 Malignant neoplasm of gum, unspecified | |  |
|  | C04 Malignant neoplasm of floor of mouth | |  |
|  | C04.0 Malignant neoplasm of anterior floor of mouth | |  |
|  | C04.1 Malignant neoplasm of lateral floor of mouth | |  |
|  | C04.8 Malignant neoplasm of overlapping sites of floor of mouth | |  |
|  | C04.9 Malignant neoplasm of floor of mouth, unspecified | |  |
|  | C05.0 Malignant neoplasm of hard palate | |  |
|  | C06 Malignant neoplasm of other and unspecified parts of mouth | |  |
|  | C06.0 Malignant neoplasm of cheek mucosa | |  |
|  | C06.1 Malignant neoplasm of vestibule of mouth | |  |
|  | C06.2 Malignant neoplasm of retromolar area | |  |
|  | C06.8 Malignant neoplasm of overlapping sites of other and unspecified parts of mouth | |  |
|  | C06.9 Malignant neoplasm of mouth, unspecified | |  |
| Oropharyngeal Cancer | C01 Malignant neoplasm of base of tongue | |  |
|  | C02.4 Malignant neoplasm of lingual tonsil | |  |
|  | C05.1 Malignant neoplasm of soft palate | |  |
|  | C05.2 Malignant neoplasm of uvula | |  |
|  | C09 Malignant neoplasm of tonsil | |  |
|  | C09.0 Malignant neoplasm of tonsillar fossa | |  |
|  | C09.1 Malignant neoplasm of tonsillar pillar (anterior) (posterior) | |  |
|  | C09.8 Malignant neoplasm of overlapping sites of tonsil | |  |
|  | C09.9 Malignant neoplasm of tonsil, unspecified | |  |
|  | C10.0 Malignant neoplasm of vallecula | |  |
|  | C10.2 Malignant neoplasm of lateral wall of oropharynx | |  |
|  | C10.3 Malignant neoplasm of posterior wall of oropharynx | |  |
|  | C10.4 Malignant neoplasm of branchial cleft | |  |
|  | C10.8 Malignant neoplasm of overlapping sites of oropharynx | |  |
|  | C10.9 Malignant neoplasm of oropharynx, unspecified | |  |

HPV, human papillomavirus; ICD-10, International Classification of Diseases (tenth revision).
