# Supplementary material for: Hospitalization and mortality burden of human papillomavirus-associated cancers among men in Ecuador: a nationwide population-based study
Source: Front Public Health. 2026 Oct 27;14:1980521. doi: 10.3389/fpubh.2026.1980521 (PMC13748293; doi:10.3389/fpubh.2026.1980521)
Supplement: Supplementary file 2 [file Table_2.docx]

**Supplemental** **Table S2.** Methods and sources for estimating HPV-attributable fraction by cancer site.

| Cancer by anatomic site | Detection method for HPV prevalence | Population | Attributable fraction (%) | Reference |
| --- | --- | --- | --- | --- |
| Anus | PCR | World | 100.0 | de Martel C 2020 |
| Oral cavity | PCR + p16INK4A | South America | 6.3 | Sichero 2022 |
| Oropharynx | PCR + p16INK4A | South America | 21.2 | Sichero 2022 |
| Larynx | PCR + p16INK4A | South America | 4.5 | Sichero 2022 |
| Penis | PCR | World | 51 | Plummer 2016 |

References:

Plummer M, de Martel C, Vignat J, Ferlay J, Bray F, Franceschi S. Global burden of cancers attributable to infections in 2012: a synthetic analysis. Lancet Glob Health. 2016;4(9):e609-16.

de Martel C, Georges D, Bray F, Ferlay J, Clifford GM. Global burden of cancer attributable to infections in 2018: a worldwide incidence analysis. Lancet Glob Health. 2020;8(2):e180-e90.

Sichero L, Tagliabue M, Mota G, Ferreira S, Nunes RAL, Castañeda CA, et al. Biomarkers of human papillomavirus (HPV)-driven head and neck cancer in Latin America and Europe study: Study design and HPV DNA/p16(INK4a) status. Head Neck. 2022;44(1):122-33.

Note: The attributable fraction in Sichero et al was calculated using data provided in the manuscript. For the oropharynx, 29/137 = 21.2%; for the oral cavity, 24/379 = 6.3%; for the larynx, 7/157 = 4.5%.
